# Transcriptome Analyses Reveal the Aroma Terpeniods Biosynthesis Pathways of *Primula forbesii* Franch. and the Functional Characterization of the *PfDXS2* Gene

**DOI:** 10.3390/ijms241612730

**Published:** 2023-08-12

**Authors:** Yin Jia, Xiancai Yin, Hongchen Yang, Yuanfen Xiang, Keying Ding, Yuanzhi Pan, Beibei Jiang, Xue Yong

**Affiliations:** College of Landscape Architecture, Sichuan Agricultural University, Chengdu 611130, China; yinxc@stu.sicau.edu.cn (X.Y.); yanghongchen@stu.sicau.edu.cn (H.Y.); yuanfenxiang@gmail.com (Y.X.); keyingding@stu.sicau.edu.cn (K.D.); scpyzls@163.com (Y.P.); sicaujbb@163.com (B.J.); yongxue@sicau.edu.cn (X.Y.)

**Keywords:** *Primula*, floral scent, transcriptome, RNA-seq, terpenoids, *DXS*, VIGs

## Abstract

*Primula forbesii* Franch. is a unique biennial herb with a strong floral fragrance, making it an excellent material for studying the aroma characteristics of the genus *Primula*. The floral scent is an important ornamental trait that facilitates fertilization. However, the molecular mechanism regulating the floral scent in *Primula* is unknown. In order to better understand the biological mechanisms of floral scents in this species, this study used RNA sequencing analysis to discuss the first transcriptome sequence of four flowering stages of *P. forbesii*, which generated 12 *P. forbesii* cDNA libraries with 79.64 Gb of clean data that formed 51,849 unigenes. Moreover, 53.26% of the unigenes were annotated using public databases. *P. forbesii* contained 44 candidate genes covering all known enzymatic steps for the biosynthesis of volatile terpenes, the major contributor to the flower’s scent. Finally, 1-deoxy-d-xylulose 5-phosphate synthase gene of *P. forbesii* (*PfDXS2*, MK370094), the first key enzyme gene in the 2-c-methyl-d-erythritol 4-phosphate (MEP) pathway of terpenoids, was cloned and functionally verified using virus-induced gene silencing (VIGs). The results showed that *PfDXS2*-silencing significantly reduced the relative concentrations of main volatile terpenes. This report is the first to present molecular data related to aroma metabolites biosynthesis pathways and the functional characterization of any *P. forbesii* gene. The data on RNA sequencing provide comprehensive information for further analysis of other plants of the genus *Primula*.

## 1. Introduction

Floral fragrances have many low-molecular-weight volatile compounds, which are secondary metabolites released by plant flowers [1,2]. Floral fragrances attract pollinating insects and repels herbivores [3], playing an important role in the reproduction of many plants. Furthermore, floral fragrances trigger a defense response after a plant is infected with a pathogen, thereby protecting the plant from damage [4,5]. Flavors and fragrances extracted from plant floral volatiles are important raw materials for many light and cosmetic industries [6]. Additionally, floral fragrances improve the ornamental value of garden plants, and aromatic plants are better than non-scented plants. The enjoyable smell enhances the visual aesthetics of their viewers; thus, floral fragrances have been known as the soul of flowers [7]. A survey of sweet pea-cut flowers in the flower market showed that floral aroma influenced consumer decisions more than color and shape [8]. Moreover, floral fragrance substances can purify the air, repel mosquitoes, and enhance health care, effectively improving people’s quality of life. These characteristics have given aromatic plants more attention, and their application prospects in development have become very attractive.

Different plants release various types of floral fragrances [2,4]. Over 2000 floral aromatic substances, mainly terpenoids, benzenoids/phenylpropanoids, and fatty acid derivatives, have been identified in 991 species belonging to 90 genera [2,9]. The rapid development of molecular biology and biochemical technologies has revealed many key enzymes and metabolic pathways controlling the production of floral fragrance metabolites. Terpenoids are the largest class of natural floral compounds in plants, are ubiquitous in plant nutrition and flower organs, and are the most diverse class of plant secondary metabolites. The terpenoid synthetic pathway includes two alternative and independent pathways: the plastidial 2-c-methyl-d-erythritol 4-phosphate pathway (MEP) in plastids, and the cytosolic mevalonate pathway (MVA) in cytoplasm. The main products of the MEP pathway are monoterpenes, diterpenes, and tetraterpenes, while the MVA pathway can synthesize sesquiterpenes and triterpenes [10,11,12]. Previous studies showed that 1-deoxy-d-xylulose 5-phosphate synthase (DXS) is the first key and rate-limiting enzyme in the MEP pathway [13,14,15,16]. In this pathway, pyruvate and glyceraldehyde-3-phosphate are substrates to generate DXS under the action of thiamine pyrophosphate (TPP) [13]. The precursor materials, biochemical reaction steps, and catalysis enzymes of the MVA and MEP pathways are completely different. However, both catalyze the formation of isopentenyl pyrophosphate (IPP) and its allylic isomer, dimethylallyl pyrophosphate (DMAPP), which are both important prerequisite substances for terpenoid formation [14]. The first plant *DXS* genes were cloned from *Arabidopsis thaliana* and *Mentha piperita*, followed by *Solanum lycopersicum*, *Pinus massoniana*, *Rosa rugosa*, and *Pelargonium* spp. [15,16,17,18]. Overexpressing the *DXS* gene can increase the production of terpenoids and improve plant tolerance to stress [19,20,21]. Therefore, *DXS* plays a key role in synthesizing terpenoids as secondary metabolites.

*Primula* L. is the largest genus in the family Primulaceae. Primrose has a high ornamental value and is well known as one of “the world’s three alpine flowers” alongside *Gentiana* (Tourn.) L. and *Rhododendron* L. The genus *Primula* has approximately 430 species worldwide, and 300 are native to China [22,23]. *Primula forbesii* Franch. is a unique biennial primrose in China, distributed in Yunnan and Sichuan provinces at an altitude of 1500–2000 m [22]. The Vilmorin seed company introduced this species into Europe in 1891, but it is rarely seen today [23]. *P. forbesii* has high ornamental value, with brightly colored flowers, a long flowering period, and produces numerous flowers. Moreover, the rich floral fragrance makes *P. forbesii* an ideal material for studying the metabolism of floral fragrances in *Primula*. For a long time, research on primrose plants has mainly focused on non-volatile substances because of their expectorant, anti-inflammatory, and antibacterial effects [24]. In recent years, studies on the content of volatile organic compound components (VOCs) have been published, but these studies are restricted to a few primrose species [25], including *P. veris*, *P. elatior*, *P. farinosa*, and *P. spectabilis* [26,27,28,29,30]. Previous studies on VOCs of *P. forbesii* in flowers at different flowering stages showed that 69 compounds, including 10 terpenoids, 21 phenylpropanoids, and 38 fatty acid derivatives, were identified in the floral components of *P. forbesii*. Among them, terpenoids contributed the most to the aroma of *P. forbesii*, and with the blooming and aging of flowers, the proportion of terpenoids increased from 52.6% at the initial flowering stage to 70.9% at the full flowering stage, then decreased to 38.6% at the final flowering stage. Linalool, a terpene compound, is a key floral component of *P. forbesii*. In addition, the diversity and content of phenylpropanoid compounds also increased with the blooming of flowers [31]. However, there are no reports on the molecular mechanisms of the floral aroma metabolism of *Primula* plants. Therefore, in order to further explore the regulatory mechanism of terpene metabolism in *Primula* plants and develop more effective plant terpene metabolism engineering, it is extremely necessary to study the molecular mechanism of floral scent biosynthesis in *P. forbesii*.

This study first established the transcriptome databases of *P. forbesii* at four different flowering stages (S1, bud stage; S2, initial flowering stage; S3, full flowering stage; and S4, final flowering stage) using RNA sequencing (RNA-seq). This technique is reliable and commonly used for studying the molecular mechanisms of various traits, especially in non-model plants that lack reference genomes [32,33,34]. RNA-seq has been used to investigate several floral fragrance secondary metabolism pathways in plants with limited genomic information, such as *Paeonia suffruticosa* [7], *Hedychium coronarium* [35], *Cymbidium goeringii* [36], *Chrysanthemum morifolium* [37] and *Freesia* spp. [32,38]. Secondly, the analysis of differentially expressed genes (DEGs) of *P. forbesii* revealed the aroma biosynthesis pathways of terpenoids. Finally, this study verified the key functions of *PfDXS2* which were screened out from the transcriptome sequencing database by gene cloning, subcellular localization, and virus-induced gene silencing (VIGs) analyses. These results identified the transcripts of *Primula* and laid an important foundation for understanding the metabolic mechanism of the floral fragrance of *Primula.* Moreover, they provide high-quality and reliable genetic resources for aroma improvement in this genus.

## 2. Results

### 2.1. Illumina Sequencing and Assembly Results

Transcriptome sequencing was performed on 12 samples from four different blooming stages of *P. forbesii* (Table S1). The total raw read means were 39,075,077 for S1, 39,031,678 for S2, 58,245,629 for S3, and 40,630,295 for S4. In contrast, the means of clean reads were 36,779,289, 36,685,813, 51,213,037, and 37,268,538 for S1 to S4, respectively. A total of 79.64 Gb clean data was obtained, each sample reaching 5.43 Gb, and the percentage of base Q30 was ≥93.49%. The mean guanine−cytosine (GC) content % in all samples was 44.95%. TRINITY assembly software (version 2.0.6) generated 51,849 unigenes (excluding the low expression transcripts) with a mean length of 1047 bp and a 1677 bp N50 length (Table 1). The length of assembled unigenes was highly diverse (300 to ≥2000 bp). The assembly generated numerous larger unigenes, including 7321 of longer than 2000 bp, 9746 of 1001–2000 bp, and 14,061 of 501–1000 bp (Figure S1).

### 2.2. Functional Annotation and Gene Expression Quantification

**Figure 1:**
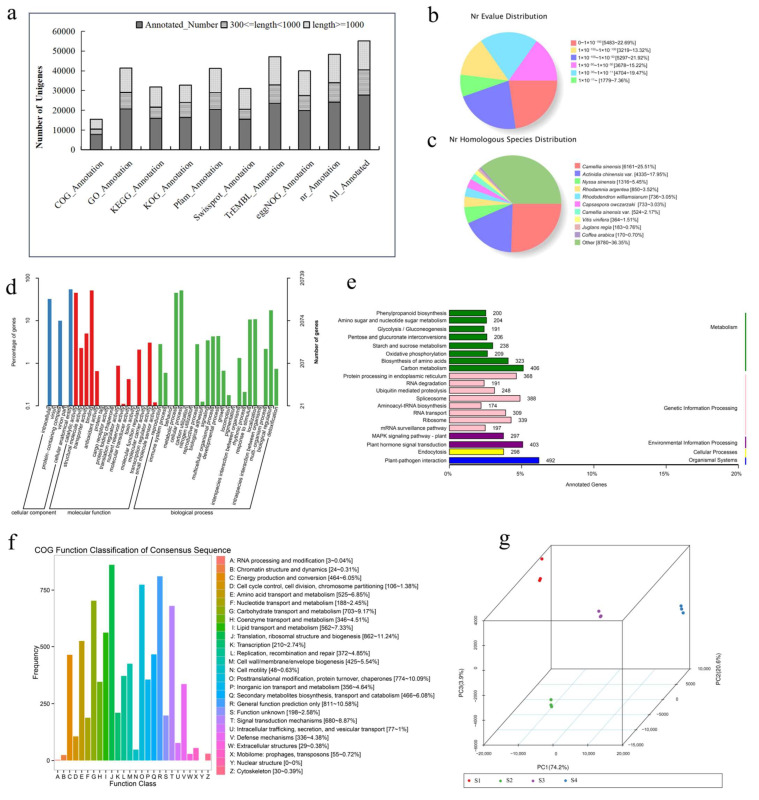
Construction of transcriptome of *P. forbesii*. (**a**) Annotation results of different databases; (**b**) E-value; (**c**) Species distribution of the top BLAST hits for each unique sequence. (**d**) The annotated results in the GO database; (**e**) Top 20 of the annotated results in the KEGG database; (**f**) The annotated results in the COG database; (**g**) A principal component analysis of the gene expression level.

Functional annotations of the unigenes against nine unigene databases identified 27,613 unigenes, accounting for 53.26% of the total unigenes, with 14,698 unigenes measuring ≥ 1000 bp. The Nr database annotated the functions of most genes (24,160), followed by TrEMBL (23,604), GO (20,739), Pfam (20,598), eggNOG (19,996), KOG (16,412), KEGG (15,943), Swiss-Prot (15,502), and COG (7737) (Figure 1a). A BLASTX search against the Nr database (with a 10^−10^ E-value cut-off) showed strong homology (0 < E-value < 10^−50^) in 57.93% of the annotated sequences, and 42.05% had 10^−50^ and 10^−11^ E-values (Figure 1b). Most of the unigene sequences were similar to the *Camellia sinensis* (25.51%), *Actinidia chinensis* (17.95%), *Nyssa sinensis* (5.45%), and *Rhodamnia argentea* (3.52%) sequences (Figure 1c).

The GO annotation results revealed 33,148 unigenes for biological process, 23,105 for molecular function, and 20,119 for cellular components (Figure 1d). KEGG pathway analysis obtained 137 enriched pathways. The top five include plant-pathogen interaction (492 unigenes), carbon metabolism (406 unigenes), plant hormone signal transduction (403 unigenes), spliceosome (388 unigenes), and protein processing in endoplasmic reticulum (368 unigenes) (Figure 1e). With respect to the pathways related to secondary metabolism, phenylpropanoid biosynthesis (200 unigenes) represented the largest group, followed by flavonoid biosynthesis (81 unigenes), terpenoid backbone biosynthesis (65 unigenes), and carotenoid biosynthesis (52 unigenes) (Table S2). Additionally, the COG classification enabled 862 unigenes to be annotated into translation, ribosomal structure, and biogenesis, 774 into post-translation modification, protein turnover, and chaperones, and 703 into carbohydrate transport and metabolism (Figure 1f). Notably, 466 unigenes were classified under secondary metabolites biosynthesis, transport, and catabolism. In addition, the unigenes were assigned to 25 functional categories in the KOG and eggNOG databases each (Figure S2). These results provide a valuable resource for gene discovery and functional research in specific *P. forbesii* biological events.

Moreover, gene expression was determined as fragments per kilobase of exon model per million mapped reads (FPKM) in the RSEM software (version 1.2.19). A principal component analysis showed a strong correlation between the performance of the four groups (S1–S4) (Figure 1g), especially in terms of biological replicates, indicating the rationality of the experimental design.

### 2.3. Analysis of DEGs

**Figure 2:**
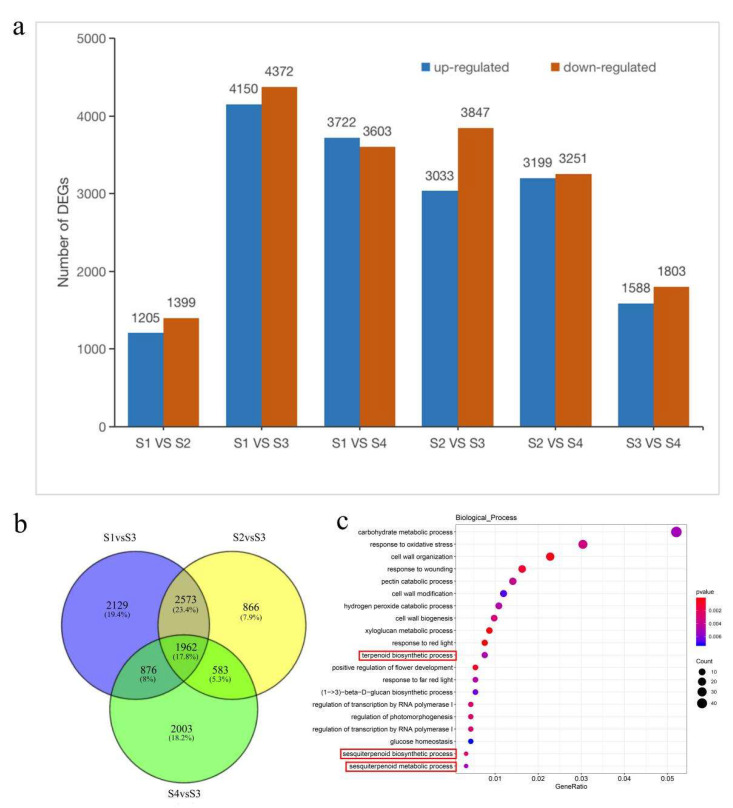
Gene expression comparisons. (**a**) Number of DEGs (up-regulated and down-regulated) in pairwise comparisons of four different flowering stages. (**b**) Venn diagram of number of DEGs in different flowering periods (S1–vs.–S3, S2–vs.–S3, S4–vs.–S3). (**c**) Top 20 of S1–vs.–S2 in biological process enrichment in GO database. The red boxes indicate the biosynthetic and metabolic process of terpenoids.

The S1–vs.–S3 combination had the highest number of DEGs (8522), with 4150 up-regulated and 4372 down-regulated genes, followed by the S1–vs.–S4 combination (7325, with 3722 up-regulated and 3603 down-regulated genes) (Figure 2a). However, the S1–vs.–S2 combination had the lowest number of DEGs (2064, with 1205 up-regulated and 1399 down-regulated genes). Few DEGs in the S1–vs.–S2 combination were related to flower opening, but this number increased sharply from S2 to the full flowering stage (S3), where it decreased rapidly in the S3–vs.–S4 combination. These results indicated that the petals have more complex biological events at the first and full flowering stages than at the budding and final flowering stages. Therefore, comparing this with the full blooming period (S1–vs.–S3, S2–vs.–S3, and S4–vs.–S3) revealed 10,992 DEGs (Figure 2b), accounting for 21.20% of the unigenes in the *P. forbesii* transcriptome. Moreover, 1962 DEGs were significantly expressed in all three developmental stages. In addition, the TrEMBL database annotated the most DEGs, followed by Nr database; the COG database annotated the fewest (Table S3).

A GO enrichment of DEGs at the S1 and S2 showed that the sesquiterpenoid metabolic process, terpenoid biosynthetic process, and sesquiterpenoid biosynthetic process were all significantly enriched (with 3, 7, and 3 DEGs, respectively) (Figure 2c). All of the 3, 7, and 3 DEGs were significantly up-related. However, no DEG from any comparisons significantly enriched the terpene synthesis process (Figure S3), indicating that the early flower opening stage is critical for terpenes synthesis.

The KEGG enrichment results showed that the DEGs significantly enriched phenylpropanoid biosynthesis, except those from S1–vs.–S3; however, terpenoid metabolism was not significantly enriched in each combination (Figure S4). Most DEGs (158) at the S1 and S3 stages are involved in the ribosome and belong to genetic information processing, followed by plant hormone signal transduction having 150 DEGs in the category of environmental information processing (Table S4). Thus, these biological processes are probably key in *P. forbesii* flowering.

### 2.4. Analysis of Genes Related to Terpenoid Biosynthesis

Terpenoids are the most important components of the VOCs in *P. forbesii* [31], leading to the analysis of KEGG annotation regarding terpenoid biosynthesis. Terpenoid backbone biosynthesis (Ko00900), monoterpenoid biosynthesis (Ko00902), sesquiterpenoid, and triterpenoid (Ko00909) revealed 44 unigenes putatively involved in terpenoid biosynthesis. The 44 unigenes belong to two distinct pathways, the plastidial MEP (30 unigenes) and the cytosolic MVA pathway (14 unigenes) (Table S5).

**Figure 3:**
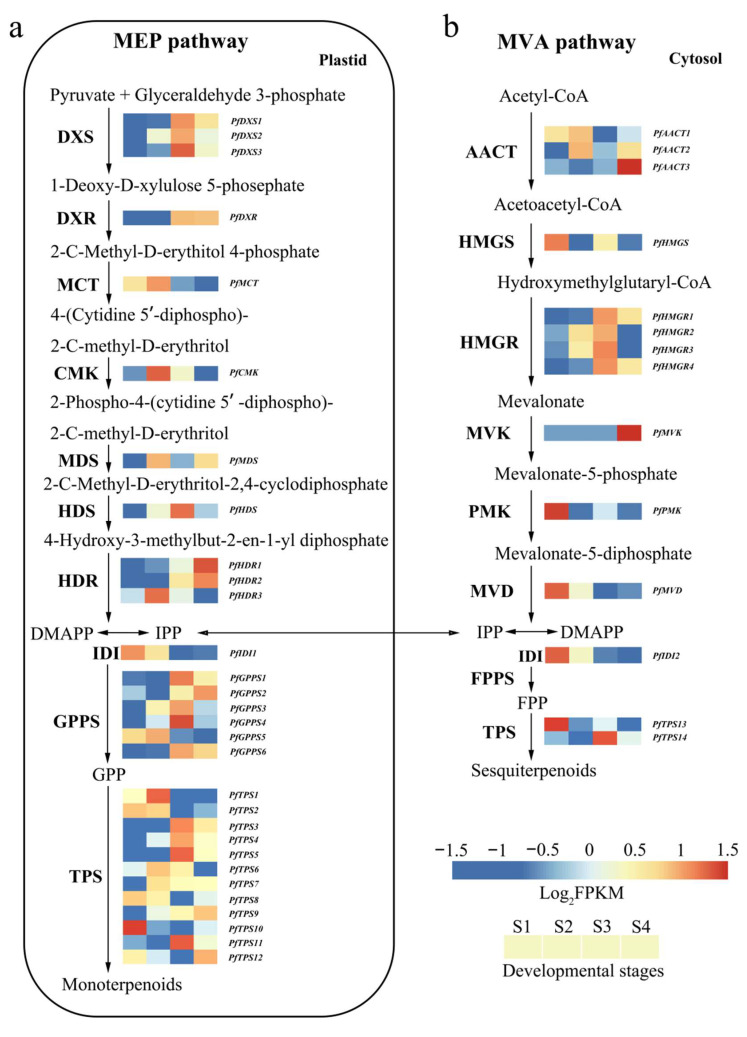
Expression patterns of terpene synthesis-related genes at different flowering developmental stages of *P. forbesii*. (**a**) MEP pathway in plastid, (**b**) MVA pathway in cytosol.

**Figure 4:**
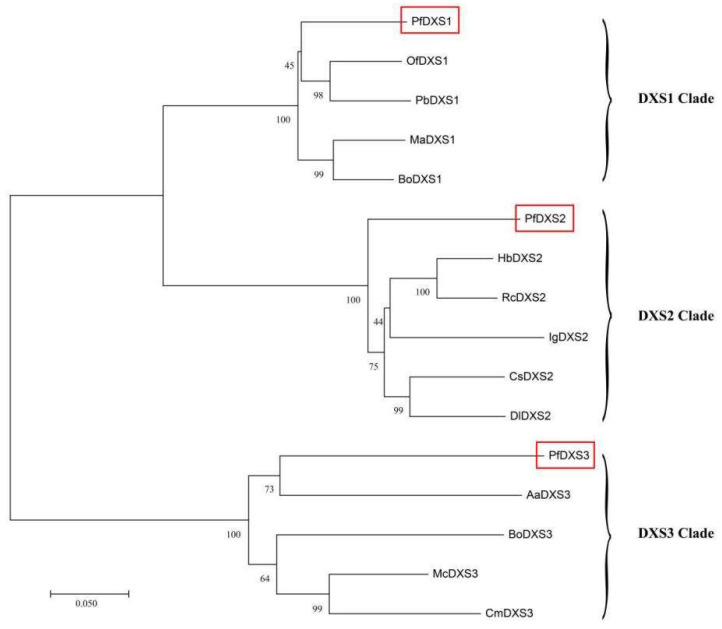
Phylogenetic tree of plant *DXSs* based on the neighbor-joining method. Three *DXSs* (designated as *PfDXS1-3*) from *P. forbesii* are marked with a red box.. The scale bar indicates 5% sequence divergence. GenBank accession numbers are shown in parentheses. Of, *Osmanthus fragrans* (AOT86855.1); Pd, *Plectranthus barbatus* (AOZ60044.1); Ma, *Melia azedarach* (KAJ4722282.1); Bo, *Bixa orellana* (AMJ39459.1); Hb, *Hevea brasiliensis* (NP_001408955.1); Rc, *Ricinus communis* (XP_002532384.1); Ig, *Impatiens glandulifera* (XP_047310950.1); Cs, *Camellia sinensis* (XP_028102700.1); Dl, *Diospyros lotus* (XP_052198114.1); Aa, *Artemisia annua* (PWA60387.1); Bo, *Bixa orellana* (AMJ39462.1); Mc, *Magnolia champaca* (ART66977.1); Cm, *Cinnamomum micranthum* f. *kanehirae* (RWR72381.1).

In the MEP pathway (Figure 3a), three unigenes supposedly encoded DXS (*PfDXS1*, *PfDXS2*, and *PfDXS3*). Phylogenetic analysis of the three *DXSs* showed that *PfDXS1* belongs to the DXS1 clade, *PfDXS2* clusters to the DXS2 clade, and *PfDXS3* to the DXS3 clade (Figure 4). Then, one gene encoding the following substances was excavated separately: 1-deoxy-D-xylulose-5-phosphate reductoisomerase (DXR), 2-C-methyl-D-erythritol 4-phosphate cytidylyltransferase (MCT), 4-diphosphocytidyl-2-C-methyl-D-erythritol kinase (CMK), 2-C-methyl-D-erythritol 2,4-cyclodipho-sphate synthase (MDS), and (E)-4-hydroxy-3-methylbut-2-enyl-diphosphate synthase (HDS). Furthermore, three unigenes were annotated as synthetic genes for 4-hydroxy-3-methylbut-2-enyl diphosphate reductase (HDR).

From the MVA pathway (Figure 3b), three unigenes were identified as acetyl-CoA C-acetyltransferase (AACT), while only one unigene was annotated as hydroxymethylglutaryl-CoA synthase (HMGS). Four unigenes were found to be hydroxymethylglutaryl-CoA reductase (HMGR). There was one gene identified as mevalonate kinase (MVK), phosphomevalonate kinase (PMK), and diphosphomevalonate decarboxylase (MVD). Interestingly, two unigenes were annotated as isopentenyl-diphosphate delta-isomerase (IDI), which converts IPP to DMAPP in a reversible reaction and regulates the equilibrium between IPP and DMAPP [39]. Protein subcellular localization prediction revealed that *PfIDI1* (MEP pathway) was localized in the chloroplast, while *PfIDI2* (MVA pathway) was localized to the cytoplasm.

The KEGG annotation results contained six unigenes that encode geranyl pyrophosphate synthase (GPPS) and farnesyl pyrophosphate synthase (FPPS) to catalyze the condensation of IPP and DMAPP in the second step of terpene biosynthesis. The condensation produced geranyl diphosphate (GPP) in plastid, and farnesyl diphosphate (FPP) in cytosol; GPP and FPP are the biosynthetic precursors of monoterpenoids and sesquiterpenes, respectively [40]. In the last step, following the formation of GPP and FPP, an array of structurally diverse cyclic and acyclic monoterpenes and sesquiterpenes were generated through the action of terpene synthase (*TPS*), which directly determines product specificity [41]. There were 14 *TPSs* identified in the currently assembled *P. forbesii* transcriptome. A BLASTP search of NCBI identified *PfTPS6-9*, a geraniol synthase gene involved in floral scent formation. Moreover, *PfTPS10–12* and *PfTPS13–14*, the orthologs of copalyl synthase and kaurene synthase, were involved in the biosynthesis of gibberellins. All the above genes involved in the terpene biosynthesis pathway require further research.

### 2.5. Analysis of Transcription Factors (TFs)

**Figure 5:**
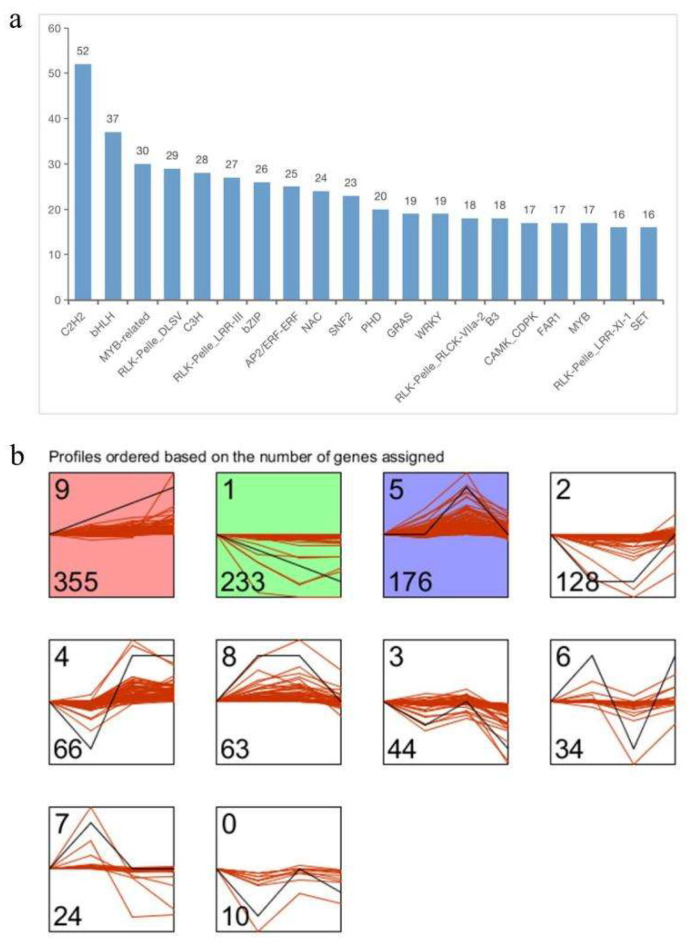
Analyses of differentially expressed TFs during flowering development. (**a**) Classification of differentially expressed TFs (Top 20). (**b**) Cluster analysis of differentially expressed TFs. All differentially expressed TFs were divided into 10 distinct temporal expression profiles using STEM software (version 1.3.13). The number in the top left hand corner is the profile ID number. Profiles are ordered based on the number of genes assigned, which is shown in the lower left hand corner. Profiles in color indicate statistical significance (*p* < 0.05) (Red, up-regulated; Purple, down-regulated; Green, up-regulated firstly then down-regulated). The red lines represent individual gene expression profiles, and the black lines show model expression profiles.

TFs regulate the expression of various genes for plant developmental and physiological processes, including plant secondary metabolism [42,43]. This study identified 2123 TFs, including transcriptional regulators (TR) and protein kinases (PK), among the 206 TF families in the PlnTFDB database. The 2123 TFs represent 4.10% of the *P. forbesii* unigenes. *C2H2* was the most abundant TF family (151), followed by *bHLH* (68), *bZIP* (57), *C3H* (55), *MYB*-related (53), *NAC* (48), and *AP2/ERF-ERF* (41) (Figure S5). A total of 1133 identified DEGs were classified into 179 transcription factor (TF) families that possibly regulate the expression of structural genes related to various biological processes of petal development. The *C2H2* family represented the largest number of differentially expressed TFs (52), followed by *bHLH* (37), *MYB*-related (30), *RLK-Pelle_DLSV* (29), *C3H* (28), *RLK-Pelle_LRR-III* (27), *bZIP* (26), and *AP2/ERF-ERF* (25) (Figure 5a).

A short time-series expression miner (STEM) cluster analysis of the differentially expressed TFs revealed ten expression profiles, including three significant expression ones (profiles 1, 5, and 9) (Figure 5b). Profiles 5, 7, 8, and 9 were further analyzed because they showed a positive correlation with the emission of volatile compounds. Profile 5 had 176 TFs distributed in 80 TF families, including *RLK-Pelle_LRR-III* (11), *C3H* (9), *SNF2* (9), *RLK-Pelle_PERK-1* (6), *RLK-Pelle_CrRLK1L-1* (5), *C2H2* (5), and *GRAS* (5) (Table S6). Moreover, 24 TFs clustered in profile 7 and included 19 TF families: three *B3*, two *RLK-Pelle_DLSV*, two *C2H2*, and two *AUX/IAA* (Table S7). There were 63 TFs in profile 8, and they were grouped into 41 TF families, including *RLK-Pelle_LRR-XI-1* (5 members), *AGC_RSK-2* (3), *RLK-Pelle_LRR-IX* (3), and *C2H2* (3) (Table S8). Furthermore, 358 TFs clustered in profile 9 and covered 106 TF families, including *RLK-Pelle_DLSV* (21), *C2H2* (17), *NAC* (12), *AP2/ERF-ERF* (11), and *WRKY* (11). (Table S9).

### 2.6. Reverse Transcription-Quantitative Polymerase Chain Reaction (qRT–PCR) Verification of DEGs

**Figure 6:**
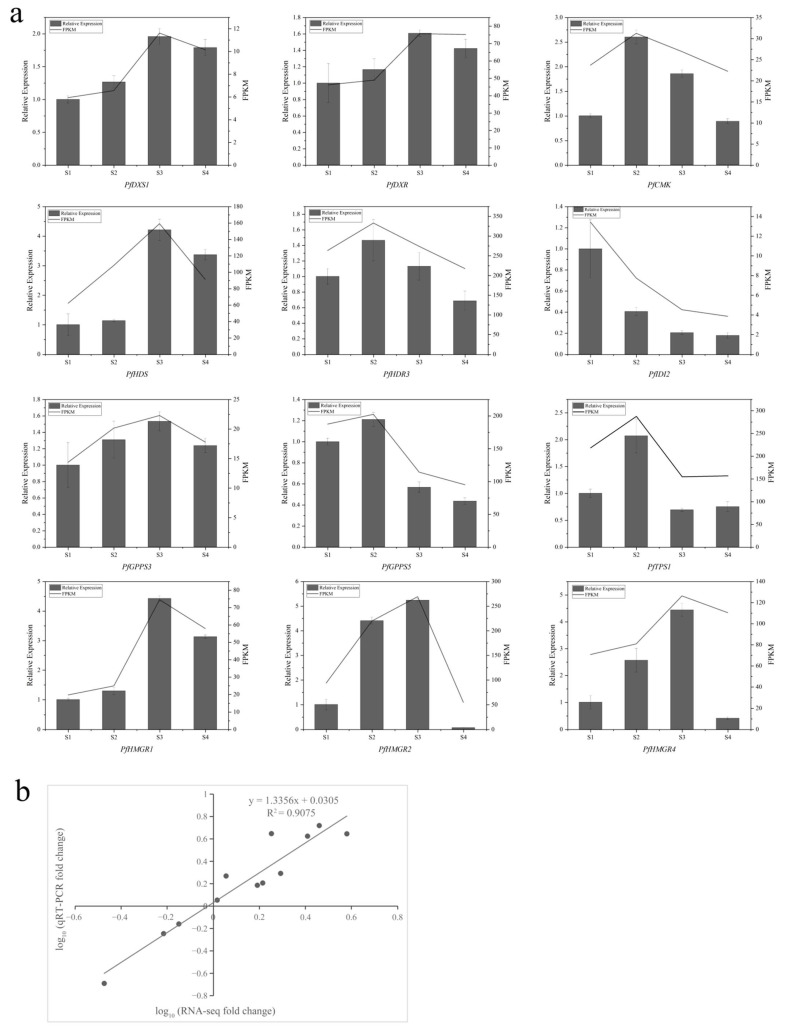
The qRT-PCR validation of DEGs. (**a**) Twelve terpene synthesis-related genes were selected for qRT-PCR determination. The expression levels of selected genes detected by qRT-PCR and RNA sequencing (RNA-seq) are shown in the same histograms. The bar chart indicates the relative expression levels of qRT-PCR (left y-axis). The line plots indicate expression levels of RNA-Seq (right y-axis). Error bars indicate the standard deviation of three independent replicates. *PfGAPDH* was used as internal reference. (**b**) Correlation analysis of fold change values obtained from RNA-seq and qRT-PCR. RNA-seq fold change refers to the ratios of FPKM values of S3 to S1 from transcriptome data, and qRT-PCR fold change is the relative quantity of S3 normalized to the expression level of S1.

Twelve DEGs related to terpenoid synthesis were selected for qRT–PCR analysis to verify the accuracy and reliability of the transcriptome data. The qRT–PCR data of these genes were highly consistent with the FPKM values in the transcriptome data from the four flowering stages of *P. forbesii* (Figure 6a). A linear regression analysis showed a 95% correlation between RNA–seq and qRT–PCR data (R = 0.95) (Figure 6b), indicating that the transcriptome data were reliable. Furthermore, the FPKM values of most of the 12 genes exhibited a low–high–low trend during flower opening–full flowering–end flowering stages, consistent with a previous study of terpenes emission in *P. forbesii* [31]. Therefore, these 12 DEGs may be involved in synthesizing terpenoids in *P. forbesii*.

### 2.7. Cloning, In-Silico Analysis, and Subcellular Localization of PfDXS2

**Figure 7:**
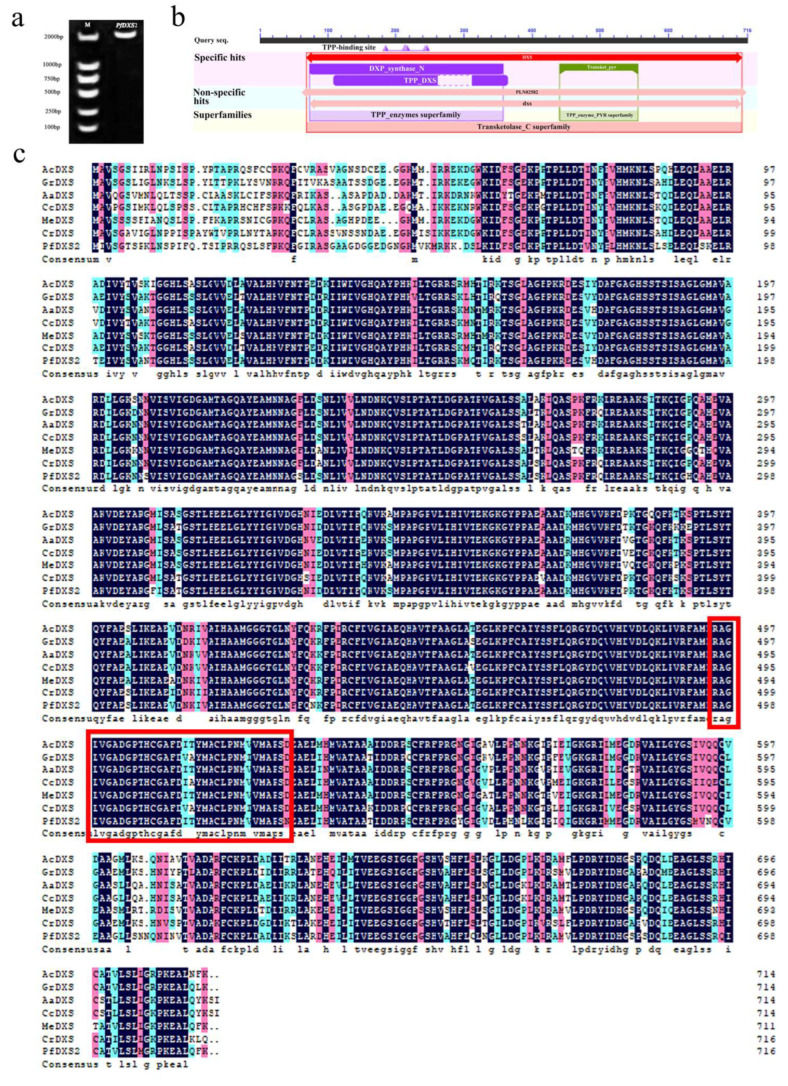
Bioinformatics analysis of *PfDXS2*. (**a**) Full-length amplification gel electrophoresis map of *PfDXS2*. (**b**) PfDXS2 protein conserved domain prediction. (**c**) PfDXS2 protein homology analysis (The red box is the transketolase domain. Ac, *Actinidia chinensis* PSS26795.1; Gr, *Gentiana rigescens* AKN79613.1; Aa, *Artemisia annua* PWA87995.1; Cc, *Cynara cardunculus* PWA87995.1; Me, *Manihot esculenta* XP_021629904.1; Cr, *Catharanthus roseus* CAA09804.2; Pf, *Primula forbesii* MK370096).

The *PfDXS2* gene discovered in the transcriptome is probably important in the biosynthesis of terpenoids in *P. forbesii* flowers. Therefore, *PfDXS2* gene sequences were cloned from *P. forbesii* (GenBank accession no. MK370094). The cloning results were 97.63% similar to the transcriptome data. ProtParam analysis showed that the ORF of *PfDXS2* is 2151 bp, and it encodes 717 amino acids (Figure 7a). The molecular weight of the encoded protein is approximately 76.8 kD with a theoretical isoelectric point of 7.34. *PfDXS2* belongs to the Transketolase_C Superfamily (69–708), having typical DXS protein domains, DXP_synthase_N (73–358 bp), and TPP_DXS (109–365 bp) (Figure 7b). PfDXS has 84.33, 81.83, 82.25, 81.69, 81.97, and 81.14% similarity with DXS proteins *Actinidia chinensis*, *Gentiana rigescens*, *Artemisia annua*, *Cynara cardunculus*, *Manihot esculenta*, and *Catharanthus roseus*, respectively (Figure 7c).

**Figure 8:**
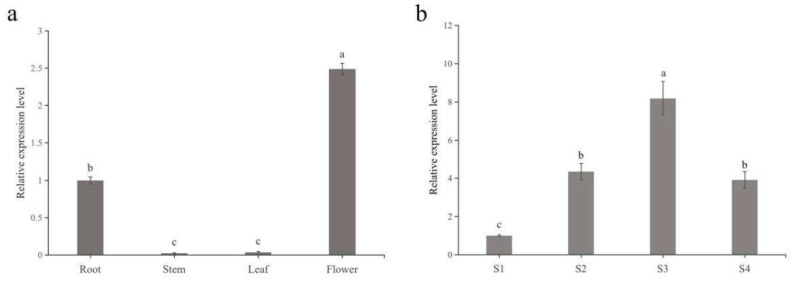
Expression patterns of *PfDXS2* in (**a**) different tissues and (**b**) different flowering stages of *P. forbesii*. The data represented the average of three independent experiments each carried out in triplicate; the error bars showed standard deviations. Different lowercase letters indicated significant differences between two treatments (*p* < 0.05).

A qRT–PCR analysis showed that *PfDXS2* was expressed in all *P. forbesii* organs (flower, leaf, root, and scape), but the expression was mainly in the flowers, followed by the roots. In contrast, *PfDXS2* expression was extremely low or negligible in the scapes and leaves (Figure 8a). The expression was 2.4, 49.0, and 35.0 times higher in the flower than in the root, scape, and leaf, respectively. The highest expression was at S3, significantly higher than at S2 and S4, and the least was at S1 (Figure 8b). These results are consistent with the release of terpenoid substances in different flowering stages of *P. forbesii* and the transcriptome expression. Therefore, *PfDXS2* may be key for terpenoid aroma biosynthesis.

**Figure 9:**
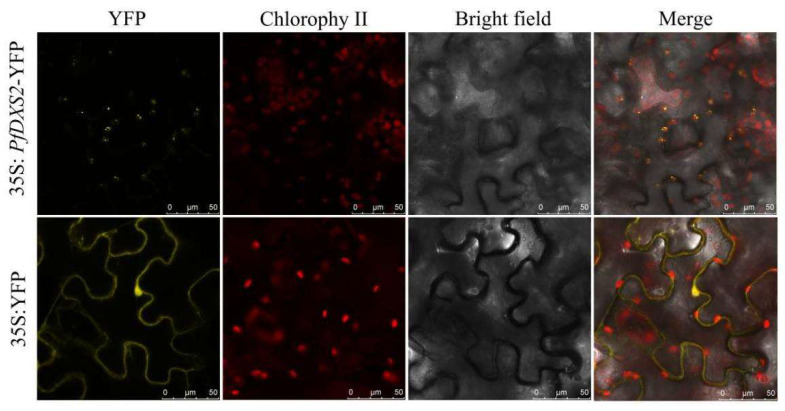
Subcellular localization of *PfDXS2*. Fluorescence signals were visualized using confocal laser-scanning microscopy. Yellow fluorescence indicates YFP, red fluorescence indicates chloroplast autofluorescence.

The subcellular localization analysis showed that the PfDXS2 yellow fluorescence was concentrated in the chloroplasts of leaf epidermal cells (Figure 9). Therefore, PfDXS2 is a plastid protein, consistent with the subcellular location of the MEP biosynthetic pathway.

### 2.8. Headspace Solid-Phase Microextraction and Gas Chromatography-Mass Spectrometry (SPME–GC–MS) of Virus-Induced PfDXS2 Gene Silencing

**Figure 10:**
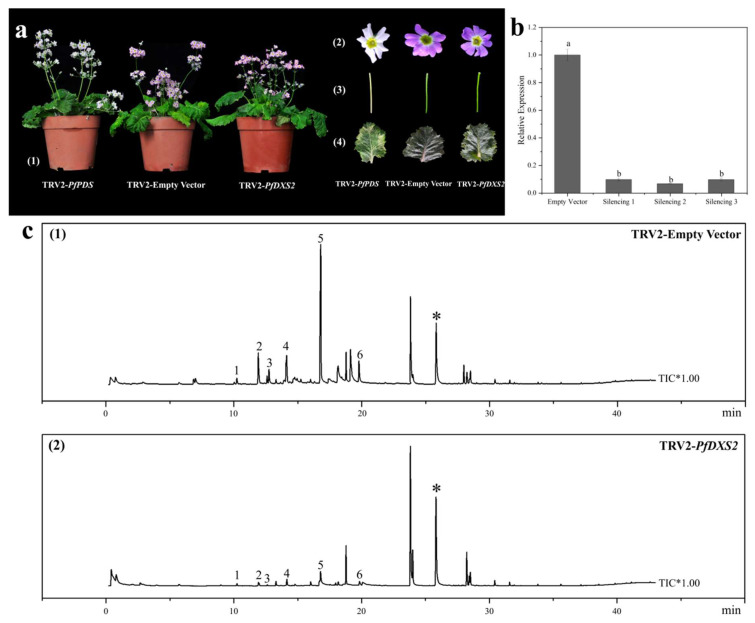
Phenotypic and main terpenoid component detection of *P. forbesii* after virus-induced gene silencing. (**a**) Phenotype of *P. forbesii* after virus-induced gene silencing. (1) Whole plant, (2) petal, (3) stem, (4) leaf. (**b**) The expression levels of the *PfDXS2* gene in empty plants and silencing plants of *P. forbesii* by qRT-PCR analysis. Lowercase letters (a, b) indicates significantly different (*p* < 0.05) based on one-way analysis of variance. (**c**) Determination of aroma components of *P. forbesii*. (1) Control treatment, (2) *PfDXS* gene silencing treatment (* Ethyl caprate as internal standard, 1 α-Pinene, 2 α-Phellandrene, 3 β-Pinene, 4 D-Limonene, 5 Linalool, 6 α-Terpineol; Unlabeled peaks were other types of VOCs or unidentified products).

Flowers in bloom from empty plants (CK) and silenced plants were subjected to qRT–PCR and SPME–GC–MS analyses (Figure 10a). The qRT–PCR results showed that silencing significantly reduced gene expression in the three plants to 9.65, 6.54, and 9.55% of the control (Figure 10b). Moreover, the six major terpenoid VOCs in *P. forbesii* were detected by SPME–GC–MS and accounted for 57.55% of the total VOCs (Figure 10c), all identified as monoterpenoids. The relative contents of the monoterpenoids were significantly low in the *PfDXS2*-silenced plants (Table 2). In particular, the relative content of linalool, the most important terpene of *P. forbesii*, significantly decreased. The mean relative content of linalool was 36.80% in the control plants, but the three *PfDXS2*-silenced plants only had 5.32, 3.23, and 13.10% mean relative contents, which were 0.14, 0.09, and 0.36 times lower than the control plants. α-Terpineol was the second most abundant terpene in the control (7.42%), and its relative contents in the gene silencing lines accounted for 14.29, 20.62, and 38.14% of the control plants. However, α-phellandrene was the third most abundant substance in the control (7.19%) and was not detected in all three *PfDXS2*-silenced plants. Moreover, the relative contents of other terpenes, D-limonene, α-pinene, and β-pinene, were significantly lower (approximately 68–95%) in the *PfDXS2*-silenced strains than in the control.

## 3. Discussion

### 3.1. Transcriptome Sequencing of P. forbesii Flowers

The genomic information on *P. forbesii* is currently lacking, so our understanding of the molecular mechanisms responsible for its floral scent is very limited. Next-generation sequencing technology is widely applied to profile the transcriptome *P. forbesii*, as applied in other non-model plants, to facilitate functional genomics research. This study utilized extensive cDNA sequences to identify genes that control floral scent and analyze floral scent biosynthesis in *P. forbesii*. Thus, the 12 cDNA libraries that were obtained during *P. forbesii* flowering were sequenced to generate 79.64 Gb of high-quality, clean transcriptome data. The Q30 of sequence reads was 93.63%, and the N50 of assembled transcriptome was 1677 bp, mirroring a high-quality transcriptome. The clean reads were assembled into 51,849 *P. forbesii* unigenes, including the 53.26% that were successfully annotated against nine public protein databases, as well as the 46.74% that showed no similarities to all the databases. Therefore, the reproductive stages of *P. forbesii* may involve many unique processes and pathways. To our knowledge, this is the first report of a large-scale transcriptome study of the genus *Primula*. The data are an important resource for unraveling specific biological processes, gene discoveries, and functional studies of *Primula* species.

Flower blooming is a highly coordinated event that ensures sexual reproduction, and blooming accompanies the enlargement of floral organs, maturity of pistil and stamen, change in flower color, and floral scent emission [44]. The five largest GO categories containing the most unigenes in this study were cellular anatomical entity, cellular process, binding, catalytic activity, and metabolic process, suggesting that cell development, various catalytic reactions, and the synthesis of metabolites underwent significant changes during flower opening. Pairwise comparison identified 2313–7540 DEGs in the four flowering stages, indicating the complex regulatory machinery involved in flower blooming. Interestingly, the KEGG enrichment results showed that phenylpropanoid biosynthesis, not terpenoid biosynthesis, was present in most flowering stage comparisons, probably because the main terpenoids were highly expressed across all the flowering stages [31]. Such an in-depth study of the mechanism of phenylpropanoid synthesis is essential for future research.

### 3.2. Volatile Terpenoid Metabolism Genes in P. forbesii

Terpenoid emission and floral scent biosynthesis have both been studied in many plants, including *Hedychium coronarium* [35], *Cymbidium goeringii* [36], *Dendrobium chrysotoxum* [45], *Prunus mume* [46], and *Rosa* spp. [47]. Thus, this study identified 44 candidate genes (30 unigenes in the plastidial MEP pathway and 14 in the cytosolic MVA pathway) that cover all known enzymatic steps in the biosynthesis of volatile terpenes. These candidate genes and their expression provide a global overview of the terpenoid formation of floral fragrances in *P. forbesii*.

This study demonstrated that *DXS*, the first enzyme of the MEP pathway, is a significant target for manipulating terpenoids biosynthesis [48,49]. Thus, understanding the function of this enzyme is valuable for potentially modulating terpene production. *P. forbesii* had three *PfDXS*, two having high expression levels. Nonetheless, all three *PfDXS* increased and decreased from S1 to S4. *PfDXS* expression was the highest in the full flowering stage, indicating that it played an important role in the monoterpene biosynthesis of *P. forbesii*. Phylogenetic analysis placed the three *PfDXS* into DXS1, DXS2, and DXS3 clades. PfDXS1 proteins possibly had a housekeeping function, *PfDXS2* was supposedly involved in secondary isoprenoid metabolism, the focus of this research, and *PfDXS3* might be related to the synthesis of some products that are essential for plant survival and required at lower levels, such as gibberellic acid and abscisic acid [35,50,51,52]. It is worth mentioning that in this study, the expression levels of *PfIDI1* and *PfIDI2* showed a high to low expression trend opposite to the biosynthesis of volatile terpenes in flowers, which was similar to the declining trend of *HcIDI2* gene expression in *Hedychium coronarium* [35]; this was probably caused by the reverse regulation of terpene synthesis by *IDI* gene in some plants. GPPS provides the branch-point intermediate GPP for terpene biosynthesis in the MEP pathway. Hsiao [53] confirmed that GPPS regulates the formation of geranyl pyrophosphate, linalool, and geraniol. This study identified six *PfGPPS* in the *P. forbesii* transcriptome, most of which had high expression and similar trends with terpenes emission, indicating the crucial role of *PfGPPS* in terpene biosynthesis. Four *PfHMGR* genes from the MVA pathway were significantly up-regulated during *P. forbesii* flowering and remained high at S3, suggesting they have crucial roles in this pathway. Plants have a wide range of *TPS* genes because *TPS* converts GPP or FPP to various terpenes via a one-step method [54]. This study identified approximately 14 *TPS* from the *P. forbesii* transcriptome, and 12 *TPSs* are involved in the MEP pathway, whereas two are involved in the MVA pathway. Therefore, this study identified more unigenes involved in monoterpene synthesis than sesquiterpene synthesis, consistent with a previous observation where monoterpene exceeded sesquiterpene contents in *P. forbesii* [31] and *Paeonia suffruticosa* [7]. However, some *PfTPS* have relatively low expression levels, such as in *Hedychium coronarium*, possibly because these genes have other functions other than volatile compound synthesis, or have completely lost their functions [54,55]. Some *TPS* genes are only expressed in specific tissues or need induction [56], indicating that there may be more *TPS* genes in *P. forbesii* that are not yet identified. Therefore, future work should consider the identification and functional verification of these *PfTPS* genes.

Some studies have verified several functional genes involved in terpenes biosynthesis. However, there is limited information regarding the transcriptional regulatory mechanism controlling floral volatile terpene biosynthesis. *MYC2*, a basic helix-loop-helix (*bHLH*) TF in *Arabidopsis*, directly regulates the formation of floral volatile terpenes [57]. Moreover, scent-related TFs, such as *MYB*, *WRKY*, *bHLH*, *bZIP*, and *AP2/ERF*, have regulatory effects on key structural genes for floral scent production [58,59,60,61,62]. This study identified 2123 TFs, including 1133 differentially expressed TFs. These TFs that correlate with the emission of volatile compounds should be the focus of future research.

### 3.3. The Gene Function of PfDXS2

This study screened *PfDXS2* from the three *PfDXS* for cloning and functional verification for the first time on floral aroma genes of the genus *Primula*. The expression of the plant *DXS* gene family is tissue-specific. For instance, the relative expression of *TcDXS* in different tissues of *Taxus chinensis* followed this trend: tender petioles > leaves > barks > roots > stems [63]. *ZbDXS* (*Zanthoxylum bungeanum*) has the highest expression in fruits, followed by leaves, and the lowest expression in flowers [64]. These studies have shown that the expression of *DXS* genes is different in different plants, and different genes in the *DXS* family are responsible for the biosynthesis of different terpenoids. In this study, *PfDXS2* expression was significantly higher in flowers than in other tissues. That expression gradually increased with the opening of flowers, peaking at the full flowering stage, consistent with its transcriptional expression and the release rate of floral aroma substances [31]. Therefore, *PfDXS2* probably plays a crucial role in aroma terpene precursor biosynthesis. Similar results were registered for *RrDXS* expression, which increased continuously during the flowering of *Rosa rugosa*, peaking at the blooming stage [17]. The subcellular localization of *PfDXS2* in chloroplasts was also important evidence that *PfDXS2* participates in the MEP pathway, consistent with the subcellular localization of *DXS* in other plants [16,65].

As the first rate-limiting enzyme of the MEP pathway, the *DXS* enzyme plays an extremely important role in regulating the terpenoid synthesis pathway. Numerous studies have shown that overexpressing the *DXS* gene increases the terpenoid content. Muñoz-Bertomeu et al. [66] showed that transferring *DXS* from *A. thaliana* into lavender (*Lavandula angustifolia*) significantly increased the content of monoterpene essential oils. VIGs analysis showed that silently expressing *PfDXS2* significantly lowers the relative content of all measured terpenes in the three plants, especially the most dominant VOC—linalool, reduced by 85.54, 91.22, and 64.40%, respectively. Other VOCs, such as α-phellandrene, α-pinene, and D-limonene, were not detected by GC–MS due to their extremely low levels in *PfDXS2*-silenced plants. Interestingly, sesquiterpene—elemene decreased by approximately 50% because MEP and MVA pathways shared a common intermediate isoprene precursor, IPP, the central precursor for the biosynthesis of all classes of terpenoids. These results confirmed the importance of *PfDXS2* in the MEP pathway for synthesizing IPP, and the amount of IPP also affected the synthesis of sesquiterpenes in the MVA pathway.

## 4. Materials and Methods

### 4.1. Experimental Materials

**Figure 11:**
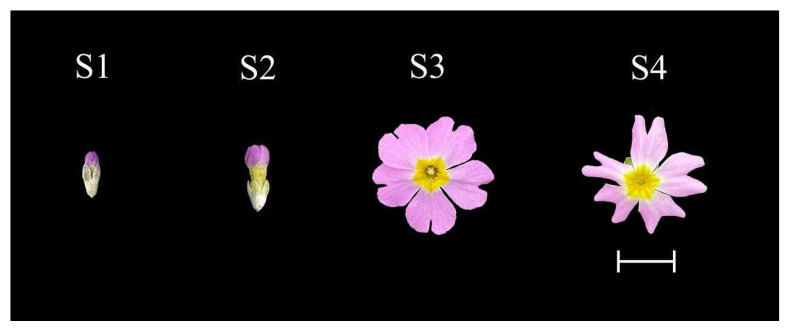
Different flowering periods of *Primula forbesii*. S1: bud stage; S2: initial flowering stage; S3: full flowering stage; S4: final flowering stage. Bar = 1 cm.

The *P. forbesii* plant materials used in this study were cultivated in the greenhouse at the College of Landscape Architecture, Sichuan Agricultural University (30°42′ N, 103°51′ E), Chengdu, Sichuan Province, China. The greenhouse growth conditions were as follows: a mixed medium of peat: perlite = 3:1 (*v*:*v*) as soil, 17 ± 3 °C daytime temperatures, 10 ± 3 °C nighttime temperatures, and ~70% relative humidity with conventional water and fertilizer management. The flowering organs of *P. forbesii* were sampled from 10 plants between 07:00 and 09:00 am on 5 January 2020 for the four flowering stages for transcriptome sequencing. The flowering stages are abbreviated S1, S2, S3, and S4. At S1, the petals were surrounded by sepals, and the sepals and petals were not expanded. At S2, the sepals were outstretched, the petals were elongated, and the flower was semi-open. At S3, the sepals and petals were fully expanded, and the petals were in full bloom. At S4, the petals were shrunk, discolored, rolled outwards, wilted, and could fall off easily (Figure 11). Flower samples were obtained simultaneously during each phase, pooled per phase, mixed, frozen in liquid nitrogen, and stored at −80 °C for transcriptome sequencing and qRT–PCR analyses.

### 4.2. RNA Extraction and Sequencing

Three biological replicates of *P. forbesii* flowers were prepared for transcriptome sequencing. Total RNA was extracted from all samples using the RNAqueousTM phenol-free total RNA kit (Ambion, Austin, TX, USA) following the manufacturer’s instructions. The RNA was purified using the RNAClean XP (Beckman Coulter, Inc., Brea, CA, USA) and RNase-Free DNase (QIAGEN, Hilden, Germany) kits. Next, the Nanodrop2000 (Thermo Fisher Scientific, Waltham, MA, USA), Qubit 2.0 (Thermo Fisher Scientific, Waltham, MA, USA), and Agilent Bioanalyzer 2100 (Agilent Technologies, Santa Clara, CA, USA) were used to detect the purity, concentration, and integrity of the RNA samples. Twelve libraries were constructed using the cDNA Synthesis kit following the manufacturer’s protocol (Illumina, San Diego, CA, USA). The sequencing library quality control was performed on the Agilent Bioanalyzer 2100 (Agilent Technologies, Santa Clara, CA, USA). The cDNA libraries were pair-end sequenced on Illumina’s HiSeq 2500 platform following the manufacturer’s guidelines.

### 4.3. De novo Assembly and Functional Annotation

The raw sequence data were filtered by removing low-quality bases (base error ratio < 0.01) and adaptor sequences. Subsequently, clean data were assembled using Trinity (version 2.0.6) following the default parameters for de novo assembly. All assembled unigenes were aligned to different databases, including the NCBI non-redundant protein (Nr), Swiss-Prot, translation of EMBL (TrEMBL), evolutionary genealogy of genes: Non-supervised orthologous groups (eggNOG), gene ontology (GO), cluster of orthologous groups of proteins (COG), eukaryotic ortholog groups (KOG), and the Kyoto encyclopedia of genes and genomes (KEGG) using BLAST. After predicting the amino acid sequence of the unigenes, HMMER software (version 3.1b2) was used to annotate the unigenes against the protein family (Pfam) database.

### 4.4. Quantification of Gene Expression Levels

TransDecoder (version r2014070464) software was used to predict the coding sequences of the unigene and their corresponding amino acid sequences. Bowtie was used to align the obtained reads to the unigene library. Finally, RNA–seq by expectation maximization (RSEM) estimated the expression using the FPKM that indicate the expression abundances of unigenes.

### 4.5. Differential Expression Analysis

The Benjamini-Hochberg method was used to correct the significant *p* values obtained by the original hypothesis test, and the *p* values were adjusted using the false discovery rate (FDR) test following the criteria: FDR < 0.05 and fold change (FC) ≥ 1.5. Functional annotation of DEGs was performed based on the expression levels of the genes in different samples following the COG, GO, KEGG, KOG, Pfam, Swiss-Prot, TrEMBL, eggNOG, and Nr annotations.

### 4.6. Analysis of the Genes That Form the Terpenoid Floral Scent

The main VOCs of *P. forbesii* are terpenoids [31]; genes that encode enzymes involved in the terpene biosynthesis pathway were analyzed in this study. The terpene biosynthesis pathway was plotted against the KEGG pathway. Furthermore, the metabolism-related genes were mined following the KEGG annotation and local BLAST search (within 10^−5^ E-value). The query sequences for local BLAST corresponded to characterized homologous genes in other plants. For complex BLAST searches, phylogenetic analysis was performed to distinguish the orthologs of corresponding functionally characterized genes. Briefly, amino acid sequences were aligned using ClustalX and used to construct the phylogenetic tree using MEGA11. Subcellular localization analysis was performed using the TargetP 1.1 Server (http://www.cbs.dtu.dk/services/TargetP/, accessed on 14 August 2020).

### 4.7. qRT–PCR Validation

Twelve DEGs closely related to terpenoid synthesis were selected for qRT–PCR expression at the four flowering stages (S1–S4) using *GAPDH* as the internal reference control. All the qRT–PCR primers were designed using Primer Premier 5 software (Table S10). The qRT–PCR assay used an Analytik Jena qTOWER 2.2 fluorescence meter (CFXCONNECT, Bio-Rad, California, USA). The reaction program involved: denaturation at 95 °C for 3 min, followed by 44 cycles of 95 °C for 5 s, and extension at 80 °C for 30 s. Finally, a melting curve analysis was performed at 95 °C for 15 s, followed by a constant increase from 60 °C to 95 °C. The qRT–PCR analyses included three independent biological replicates, each with three technical replications. The relative expression of candidate DEGs was analyzed using the 2^−ΔΔCT^ method [67].

### 4.8. Cloning, In-Silico Analysis, and qRT–PCR Validation of the PfDXS2 Gene

We analyzed the first rate-limiting enzyme in the MEP pathway, the *DXS* gene (named *PfDXS2*), from the transcriptome data to verify its role in the synthetic metabolic pathway of terpenes in *P. forbesii*. The total RNA extracted from flowers was reverse transcribed into cDNA and used to clone the full-length *PfDXS* gene (qDXS-F:CAGGACAAGCGTATGAGGCGATG; qDXS-R:CAGGAGTTGCAGGACCATCAAGTG) using primers designed from both ends of the gene. The TA cloning method was used to amplify the full length of the *PfDXS* gene, gel purification, recovery, and connection to the T vector. The construct was transformed into *Escherichia coli* competent cells overnight, and the positive PCR products were sequenced accordingly. The sequencing results were compared with the transcriptome data and in silico analysis. The open reading frame (ORF) of *PfDXS2* was predicted using the ORF finder (https://www.ncbi.nlm.nih.gov/orffinder, accessed on 16 September 2020). Next, the conserved domain of the *PfDXS2*-encoded protein was inferred from the NCBI website (https://www.ncbi.nlm.nih.gov/Structure/cdd/wrpsb, accessed on 20 September 2020), and DNAMAN 8.0 software compared the homologous sequences.

Total RNA was extracted from different tissues (flowers, roots, scapes, and leaves) sampled from different (S1–S4) *P. forbesii* flowering stages following the method above. The expression of *PfDXS2* in different tissues at different flowering stages was explored using the qRT–PCR technique, involving three biological and three technical replicates each.

### 4.9. Subcellular Localization of the PfDXS2 Gene

The amplified product of *PfDXS2* was inserted into the EcoRI/SpeI site of p131-35S-YFP to form a subcellular localization recombinant plasmid, p131-DXS2-YFP for DXS2-YFP fusion expression, with the p131-35S-YFP empty vector as the control. Then, the two vectors were transferred into Agrobacterium-competent GV3101 and injected into 4–5 leaves of *Nicotiana benthamiana*. A laser confocal microscope (TCS SP8, Leica, Weztlar, Germany) was used to observe the YFP fluorescence and chlorophyll autofluorescence at 514 nm excitation wavelength, and emission wavelengths of 530–580 nm and 650–750 nm were noted, respectively.

### 4.10. VIGs for Functional Analysis of the PfDXS2 Gene

The VIGs technology was applied to obtain *PfDXS2*-silenced plants of *P. forbesii* following the Fu et al. [68] method, using tobacco rattle virus (TRV) as the VIGS vector. Specifically, an infusion solution containing 200 μmol/L acetosyringone (AS), 10 mmol/L MgCl_2_, and 10 mmol/L ethane sulfonic acid buffer (MES) was mixed with a bacterial solution (of 1.0 OD600) containing TRV1 and *PfDXS2*–TRV2. The plants of *P. forbesii* were infected through abaxial leaf injection using the mixed bacterial solution. Next, a PCR was performed on the leaves of the bacteria-treated plants using the primers for the TRV virus vector. Virus vectors of TRV1 and TRV2 were detected in both the albino and no-load groups.

After blooming, the flowers of the three albino and no-load plants were selected randomly to analyze the expression of *PfDXS2* using qRT-PCR and the VOCs using SPME–GC–MS. Fresh flowers (approximately 15 flowers, ~0.6 g) were collected from 07:00 am to 09:00 am and immediately placed in a clean 20 mL headspace vial (Agilent, CA, USA). The headspace vial containing the sample was incubated at 50 °C for 5 min, and aged thrice on the extraction head (1.1 mm, CARBON–WR/PDMS) at 250 °C for 2 min each. The extract was adsorbed at 50 °C, with a constant temperature for 30 min; the extraction head was pulled out and quickly inserted into the GC–MS (TQ8050, Shimadzu, Japan) injection port in the pre-operation state. The solution was desorbed at 250 °C for 2 min, and finally, a 30 m × 0.25 mm × 0.25 μm InertCap Pure–WAX capillary column (Shimadzu, Japan) was used for VOCs analysis. Helium was the carrier gas at 3.0 mL∙min^−1^ linear velocity. The initial oven temperature was 50 °C for 5 min, and was increased by 10 °C∙min^−1^ up to 250 °C where it was held for 2 min. The mass spectra were taken at 70 eV (in EI mode) with a 39–280 amu mass scan range. The ion source and transfer line temperatures were 200 °C and 250 °C, respectively. Ethyl caprate was the internal standard, and each experiment was replicated three times. The compounds were identified by comparing the mass spectra and retention times with the NIST11 library. Furthermore, the relative content of the individual volatile components was calculated from the peak area, and the sum of the identified volatiles was considered the total volatile content.

## 5. Conclusions

This is the first known report showing the molecular data related to the aroma metabolite biosynthesis pathways of the genus *Primula* and the functional characterization of aroma genes in *P. forbesii* plants. RNA–seq revealed the molecular mechanisms of floral terpenoid biosynthesis pathways in four flower developmental phases in *P. forbesii.* Finally, we cloned a full-length *PfDXS2* from the DEGs. Virus-induced *PfDXS2* gene silencing significantly decreased the relative content of terpenoids. These findings lay a foundation for revealing the synthetic pathway of the floral fragrance of *P. forbesii* and provide useful molecular information for improving the quality of *Primula* fragrance in the near future. However, since only the first key and rate-limiting enzyme in the terpenoid MEP metabolic pathway was cloned and functionally validated in this study, the functions of other key genes of terpeniod metabolism need to be further explored. In addition, phenylpropanoid compounds are the second important component of the floral composition of *P. forbesii*, and their biosynthetic mechanisms need to be explored in the future to improve the molecular regulatory mechanism of the metabolism of the floral components of *P. forbesii*.

## Figures and Tables

**Table 1:** Statistical table of assembly results of *P. forbesii* transcriptome.

Samples	S1	S2	S3	S4	Total
Total raw reads	39,075,077	39,031,678	58,245,629	40,630,295	176,982,679
Total clean reads	36,779,289	36,685,813	51,213,037	37,268,538	161,946,677
GC content (%)	45.10	44.62	45.21	44.86	—
Q30 (%)	93.49	93.58	93.70	93.76	—
Total clean data (Gb)	79.64				
Total number of unigenes	51,849				
Total length of unigenes (bp)	54,302,128				
Mean length of unigenes (bp)	1047				
N50 length (bp)	1677				

**Table 2:** Comparative analysis of terpenoid floral components in the control and *PfDXS*-silencing plants of *P. forbesii* ^1^.

Components	CAS	Retention Time (min)	Retention Index	Relative Amount (%)			
				Gene Emptying (CK)	Gene Silencing		
α-Pinene	7785-70-8	10.233	943	1.31 ± 0.19 a	/	0.13 ± 0.02 b	/
α-Phellandrene	99-83-2	11.909	948	7.19 ± 0.68 a	/	/	/
β-Pinene	127-91-3	12.741	969	2.05 ± 0.63 a	0.11 ± 0.06 b	0.37 ± 0.16 b	0.50 ± 0.07 b
D-Limonene	5989-27-5	14.100	1018	2.78 ± 0.27 a	/	0.89 ± 0.33 b	/
Linalool	78-70-6	16.786	1082	36.80 ± 6.96 a	5.32 ± 0.54 bc	3.23 ± 0.42 c	13.10 ± 1.55 b
α-Terpineol	10482-56-1	19.825	1142	7.42 ± 2.89 a	1.06 ± 0.17 b	1.53 ± 0.11 b	2.83 ± 0.32 b

^1^ Data are expressed as means ± S.D. (n = 3). Lowercase letters (a, b, c) indicates significantly different (*p* < 0.05) based on one-way analysis of variance. “ / ” indicates “not detected”.

## Data Availability

All data generated and analyzed during this study are included in the published article.

## Supplementary Materials

The following supporting information can be downloaded at: https://www.mdpi.com/article/10.3390/ijms241612730/s1.
