# Supplementary material for: Transcriptome Analyses Reveal the Aroma Terpeniods Biosynthesis Pathways of Primula forbesii Franch. and the Functional Characterization of the PfDXS2 Gene
Source: Int J Mol Sci. 2023 Aug 12;24(16):12730. doi: 10.3390/ijms241612730 (PMC10454305; doi:10.3390/ijms241612730)
Supplement: Supplementary file 1 [file ijms-24-12730-s001.zip › Figure S.pdf]

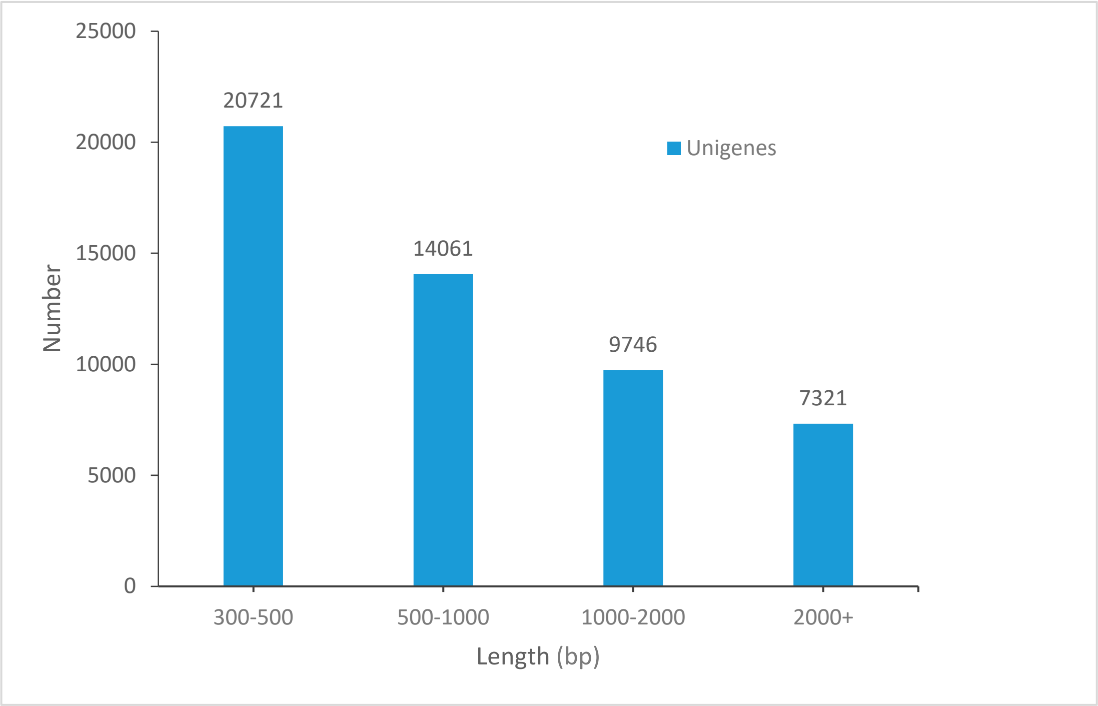

Figure S1. Length distribution of assembled unigenes

a

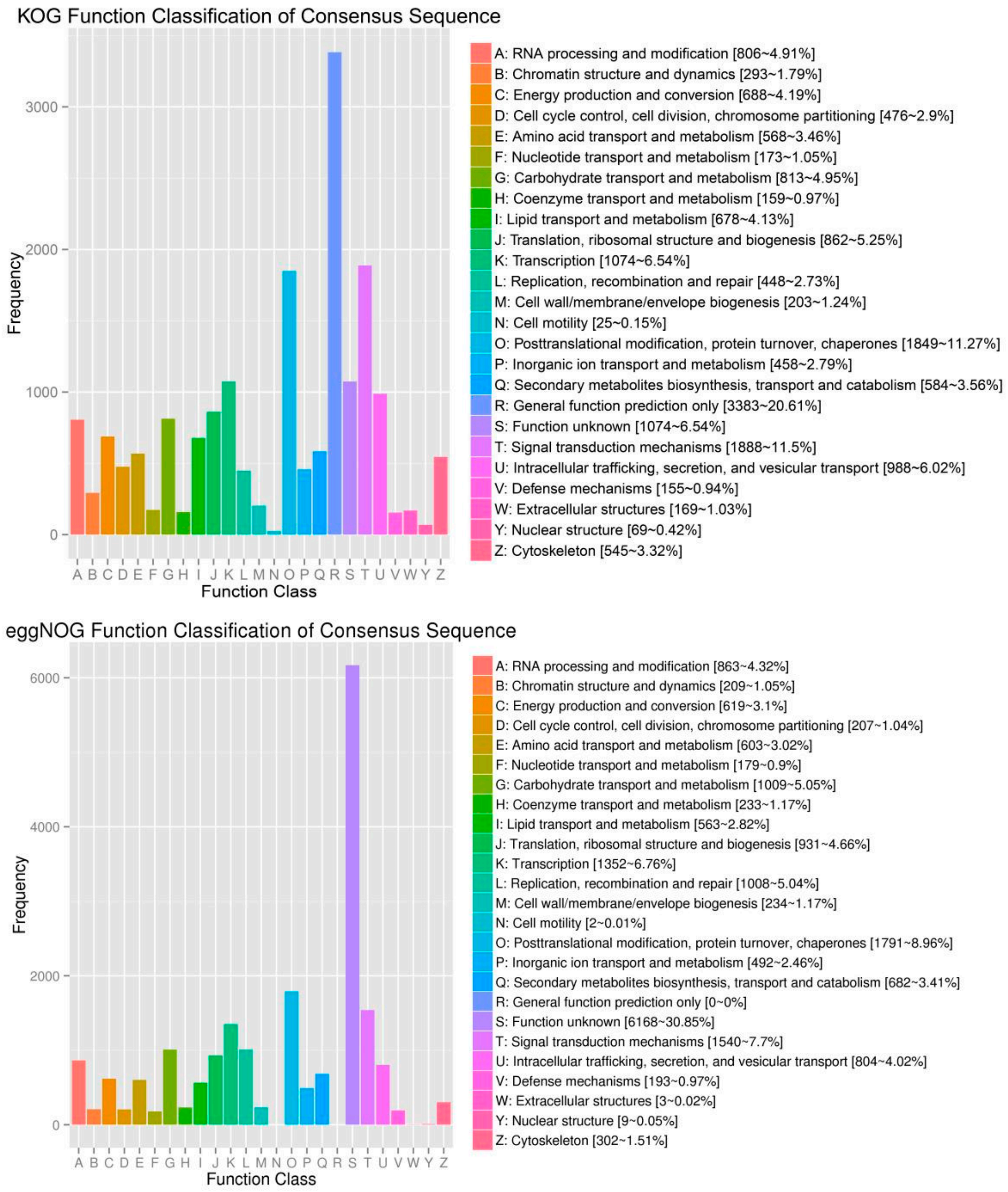

b

Figure S2. Function classification of consensus sequence in **a** KOG database and **b** eggNOG database

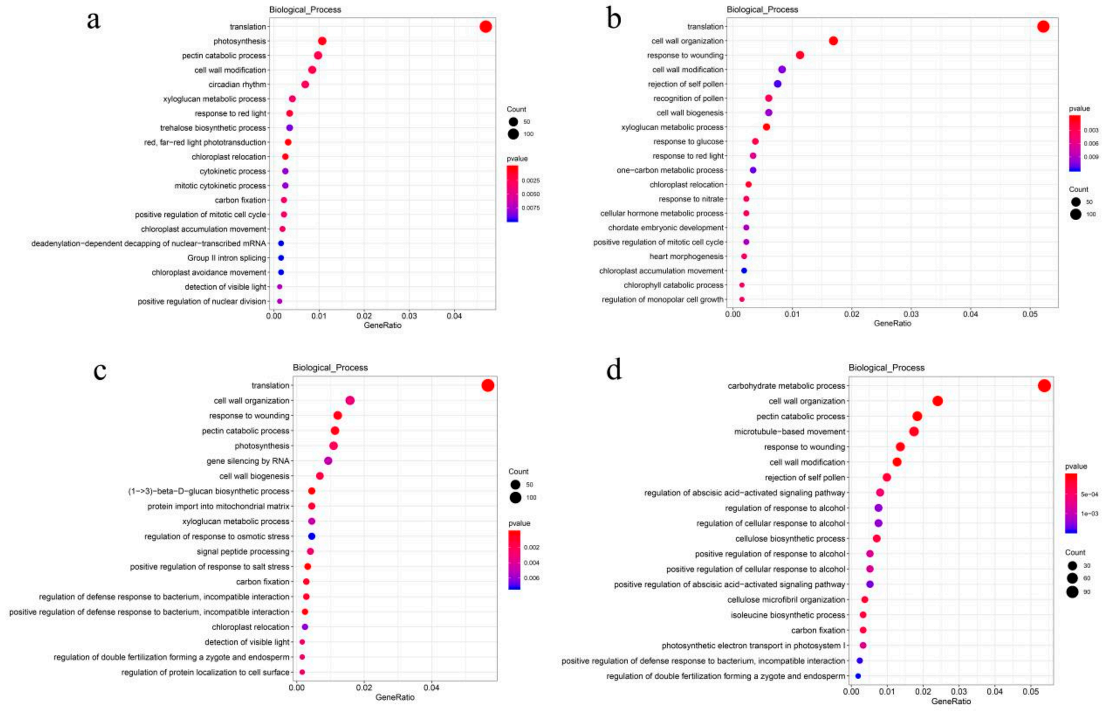

Figure S3. GO enrichment analysis of the DEGs identified in **a** S1 vs. S3, **b** S1 vs. S4, **c** S2 vs S3, d S3 vs. S4.

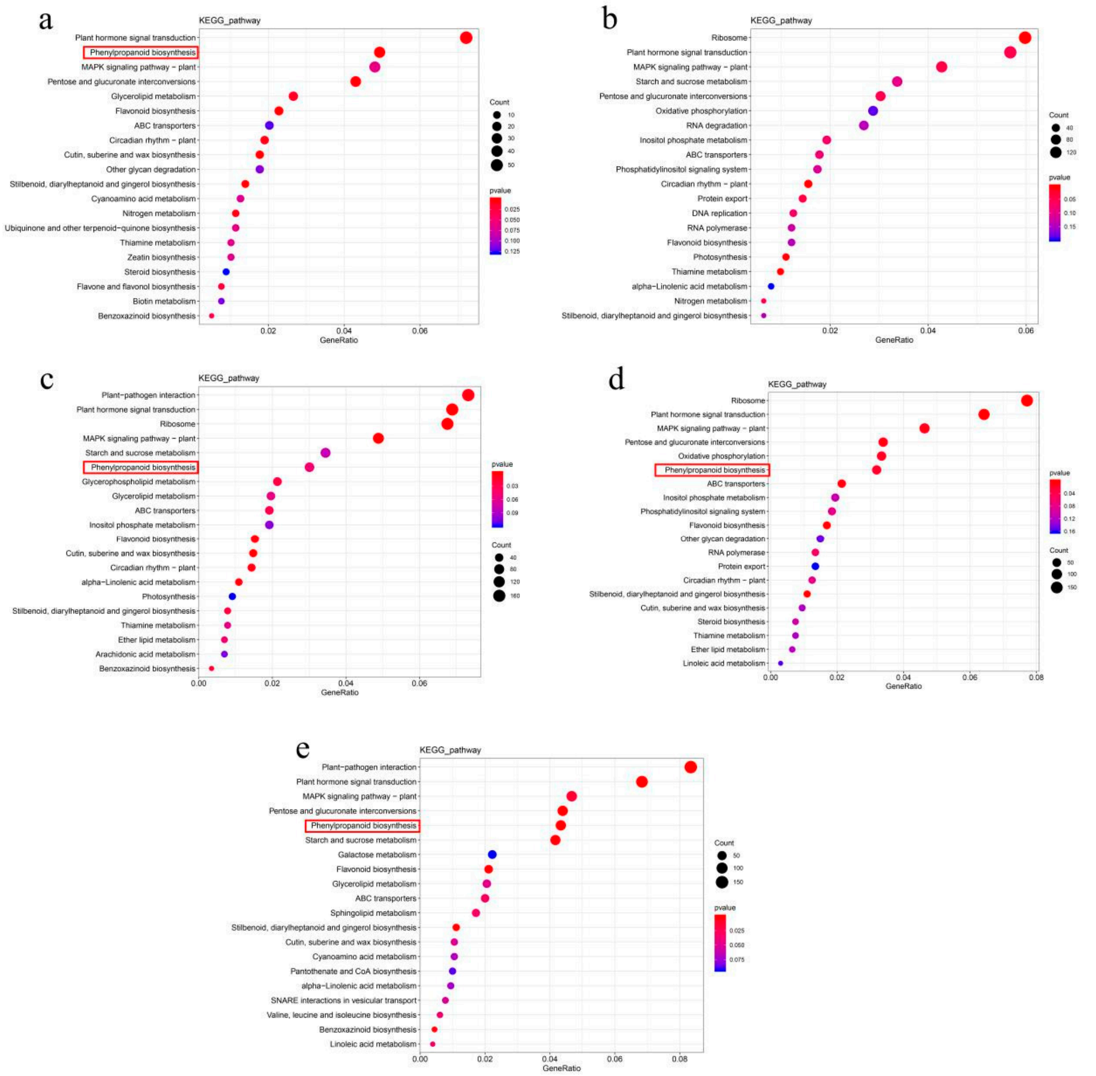

Figure S4. KEGG enrichment analysis of the DEGs identified in **a** S1 vs. S2, **b** S1 vs. S3, **c** S1 vs. S4, **d** S2 vs. S3, and **e** S3 vs S4.

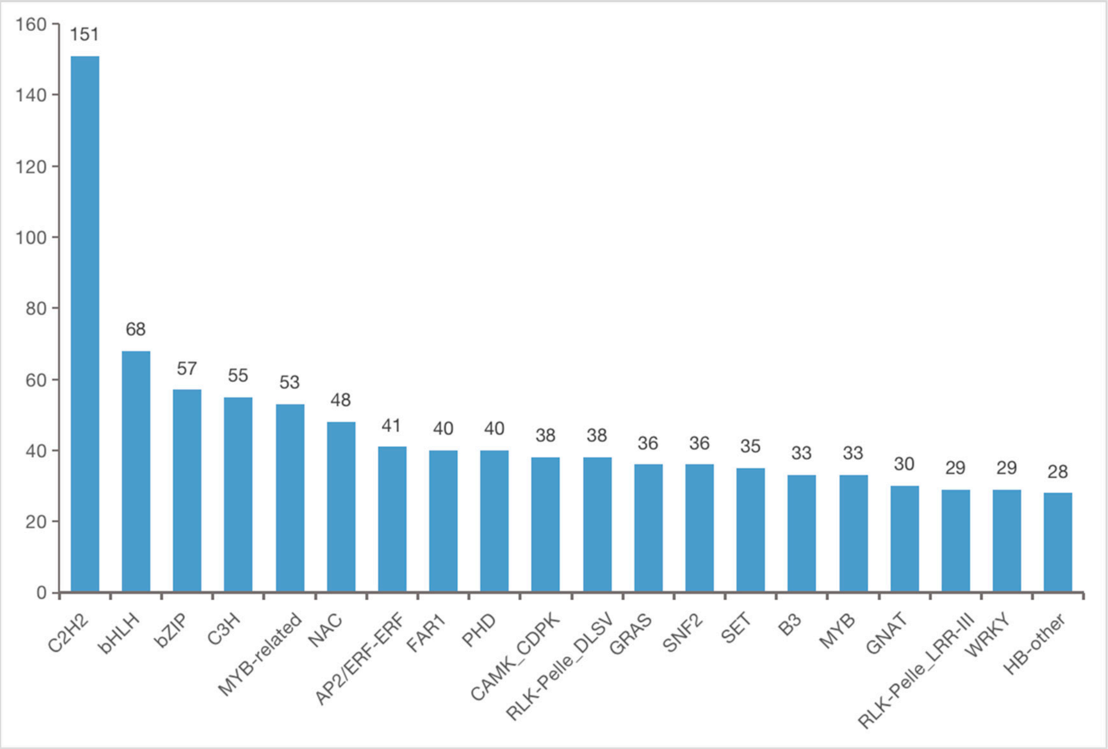

Figure S5. Top 20 of classification of all TFs
